# Differential Secretome Profiling of Human Osteoarthritic Synoviocytes Treated with Biotechnological Unsulfated and Marine Sulfated Chondroitins

**DOI:** 10.3390/ijms21113746

**Published:** 2020-05-26

**Authors:** Rosita Russo, Valentina Vassallo, Antonietta Stellavato, Mariangela Valletta, Donatella Cimini, Paolo Vincenzo Pedone, Chiara Schiraldi, Angela Chambery

**Affiliations:** 1 Department of Environmental, Biological and Pharmaceutical Sciences and Technologies, University of Campania Luigi Vanvitelli, 81100 Caserta, Italy; rosita.russo@unicampania.it (R.R.); mariangela.valletta@unicampania.it (M.V.); paolovincenzo.pedone@unicampania.it (P.V.P.); 2 Department of Experimental Medicine, School of Medicine, University of Campania Luigi Vanvitelli, 80035 Naples, Italy; valentina.vassallo92@gmail.com (V.V.); antonietta.stellavato@unicampania.it (A.S.); Donatella.cimini@unicampania.it (D.C.); chiara.schiraldi@unicampania.it (C.S.)

**Keywords:** marine chondroitin, biotechnological unsulfated chondroitin, secretome, osteoarthritis, mass spectrometry

## Abstract

Symptomatic slow-acting drugs (SYSADOA) are increasingly used as effective therapies for osteoarthritis, representing an attractive alternative to analgesics or non-steroidal anti-inflammatory drugs to relieve disease symptoms. Pharmaceutical preparations of chondroitin sulfate, derived from animal sources, alone or in combination with glucosamine sulfate, are widely recognized for their beneficial effect on osteoarthritis treatment. A growing interest has also been devoted to understanding the molecular mechanisms modulated by SYSADOA using -omic strategies, most of which rely on chondrocytes as a model system. In this work, by using an integrated strategy based on unbiased proteomics and targeted cytokine profiling by a multiplexed protein array, we identified differences in the secretomes of human osteoarthritic synoviocytes in response to biotechnological unsulfated, and marine sulfated chondroitins treatments. The combined strategy allowed the identification of candidate proteins showing both common and distinct regulation responses to the two treatments of chondroitins. These molecules, mainly belonging to ECM proteins, enzymes, enzymatic inhibitors and cytokines, are potentially correlated to treatment outcomes. Overall, the present results provide an integrated overview of protein changes in human osteoarthritic synoviocytes secretome associated to different chondroitin treatments, thus improving current knowledge of the biochemical effects driven by these drugs potentially involved in pathways associated to osteoarthritis pathogenesis.

## 1. Introduction

Osteoarthritis (OA), a leading cause of disability in adult individuals, is a degenerative joint disease characterized by a multi-factorial etiology including obesity, aging and heredity. The major OA consequences include the progressive destruction of articular cartilage and the formation of osteophytes associated with variable degrees of synovial membrane inflammation [1]. In particular, synovitis represents a key factor in OA pathophysiology by reflecting the structural progression of the disease through the production of several pro-inflammatory mediators such as IL-1β and TNF-α [2]. Current pharmacological treatments for OA are mainly symptom-focused and include combination therapies based on the analgesics or non-steroidal anti-inflammatory drugs (NSAIDs) administration and non-pharmacological methods (i.e., physical exercises and physiotherapy). However, due to the numerous side effects deriving from the prolonged use of NSAIDs, chondroitin sulfate (CS) and glucosamine (GlcN) have been proposed as anti-osteoarthritis agents since 1990s. CS is a glycosaminoglycan made of 4)-β-GlcA-(1→3)-β-GalNAc-(1 disaccharide repeating units (GlcA = glucuronic acid, GalNAc = N-acetyl-galactosamine) with molecular weights (Mw), types and grades of sulfation related to animal tissues or age [3]. With respect to the high Mw (30–80 kDa) CS from marine organisms (e.g., shark, skate, etc.), CS from terrestrial animal cartilages (e.g., pig, bovine, chicken) has a lower Mw (14–26 kDa) and a different sulfation pattern [4]. Specifically, highly purified pharmaceutical grade preparations of CS are included within the “symptomatic slow-acting drugs for osteoarthritis” (SySADOA) class [5,6]. Accordingly, SySADOA treatments have been recommended in the guidelines published by the Osteoarthritis Research Society International (OARSI) for the management of knee OA [7,8,9] and by the European League Against Rheumatism (EULAR) for the management of hip and knee OA [5,10,11].

Furthermore, based on the similarity of the microbial capsular polysaccharides to glycosaminoglycan, bacteria proved to be potential alternative non-animal sources of glycosaminoglycan-derived products [12,13]. Indeed, high purity and endotoxin-free microbial-derived unsulfated biotechnological chondroitin (BC) has been previously obtained through a patented biotechnological production process [14]. Therefore, the use of BC for medical applications could be an important alternative to extractive CS in order to overcome on one side potential contaminations issues and, on the other increasing ethical and religious concerns [15]. Recently, BC proved to be more effective in chondrogenic phenotype preservation and in the reduction of the inflammatory response in IL-1β-treated chondrocytes with respect to CS-treated cells [16]. Several efforts have been also directed to a deep understanding of cellular response to different CS treatments at molecular level. Recently, the advent of novel high throughput technologies has opened new perspectives in osteoarthritis research by mass spectrometry-based proteomic approaches [17]. In addition, cell secretome profiling has become an active area of research for the high potential in biomarker discovery with diagnostic and/or prognostic significance [18]. In particular, due to the pivotal importance of the microenvironment in OA, several studies have addressed the characterization of secretomes from human articular chondrocytes [19,20]. In addition, proteome and secretome studies investigate the effects driven by bovine/pig CS [21,22] or by combined formulations of chondroitin/glucosamine [23,24].

In the present work, an in-depth secretome analysis of primary cultures of OA synoviocytes treated with a pharmaceutical-grade marine CS has been performed by label-free quantitative high-resolution mass spectrometry (MS). Moreover, to compare the modulation effects of treatments with biotechnological and extractive agents on OA synoviocytes protein secretion, the same approach has been applied to determine the OA synoviocytes secretome profiles following treatment with pharma-grade unsulfated biotechnological chondroitin. Collectively, our results provide a detailed characterization of the human osteoarthritic synoviocytes response to biotechnological unsulfated and marine sulfated chondroitins, identifying candidate proteins potentially associated to treatment outcome. These mainly belong to ECM proteins, enzymes, enzymatic inhibitors, cytokines, and growth factors. Although an overall similar trend in protein changes was driven at the extracellular level by CS and BC, a distinct regulation response was observed for a subset of proteins, paving the way to the development of formulations using unsulfated biotechnological chondroitin for counteracting OA-related pathological processes.

## 2. Results

### 2.1. Secretome Profiling of the Synoviocytes Response to BC and CS Treatment

To investigate the activation of extracellular signaling pathways following different chondroitin treatments, we performed a global label-free quantitative high-resolution mass spectrometry-based secretome analysis of OA synoviocytes treated with a marine CS and an unsulfated biotechnological (BC) chondroitin.

**Figure 1:**
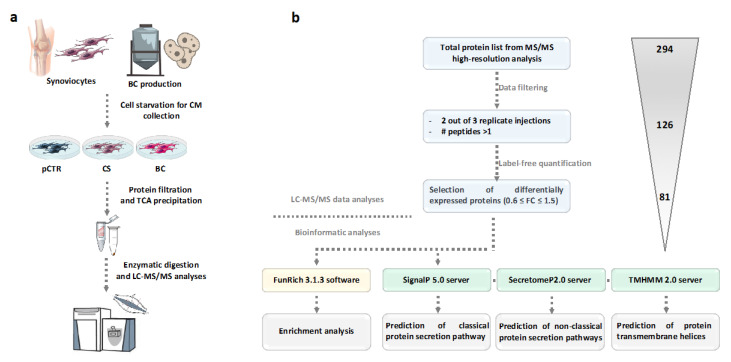
Flowchart of the workflow applied for secretome analysis of human OA synoviocytes treated with a marine CS and an unsulfated biotechnological (BC) chondroitin: (**a**) Workflow used for the high-resolution LC-MS/MS analyses of CS-/BC-treated synoviocytes secretomes. (**b**) Schematic analysis pipeline showing main sequential steps for LC-MS/MS data processing and bioinformatic analyses performed on filtered proteomic data.

Supernatants of CS-/BC-treated synoviocytes and untreated control (pCTR) were collected and concentrated by ultrafiltration and subjected to TCA precipitation. Following reduction and alkylation steps, samples were treated with trypsin and analyzed by LC-MS/MS [25] (Figure 1a).

Using high-resolution MS and the Proteome Discoverer proteomics software package for computational analysis and label-free quantitation, we compared relative protein abundances in conditioned media of CS-/BC-treated OA synoviocytes with respect to untreated cells. In particular, for each treatment replicate, we required a minimum of two replicates and a at least two peptides per protein in at least one out of three conditions for the identification to be considered reliable. According to these criteria, a total of 126 proteins were identified across the three conditions (Supplementary Table S1). From this list, we then extracted a subset of 81 proteins whose relative expression levels changed 1.5-fold or more (in any direction) in both (Table 1) or one (Table 2) of the treatment conditions with respect to untreated OA synoviocytes. We also identified several other known cartilage elements, such as cartilage oligomeric matrix protein (*COMP*) and aggrecan, that were not considered because they did not fulfil the filtering criteria used in this study.

### 2.2. Cellular Localization of the Differentially Expressed Proteins in the OA Synoviocytes Secretome

**Figure 2:**
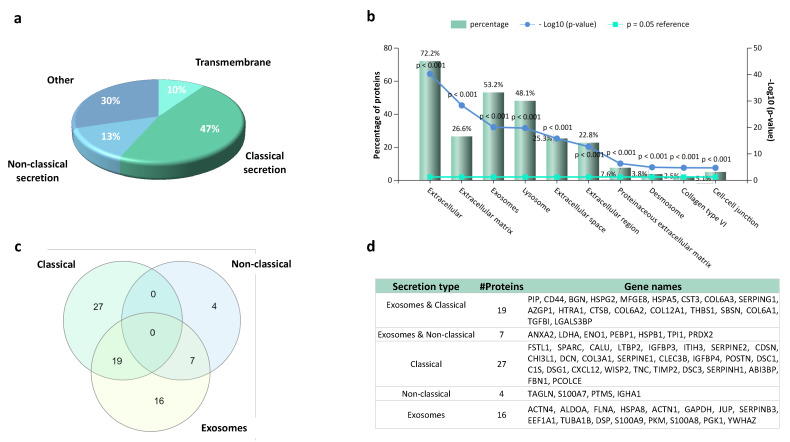
Overview of secretory pathway prediction results for differentially expressed proteins in the OA synoviocytes secretome following BC and CS treatments: (**a**) Pie chart representing the secretory pathway prediction for differentially expressed proteins by SignalP (classical secretion), SecretomeP (non-classical secretion) and TMHMM (transmembrane) servers. (**b**) Bar chart of the significantly enriched cellular components GO terms obtained for differentially expressed proteins in CS-/BC-treated synoviocytes secretomes. The number of proteins belonging to the enriched gene ontology term and the *p*-values are reported on the graph. (**c**) Venn diagram showing the overlapping of proteins secreted via classical/non-classical secretion pathways and by exosomes. (**d**) Details of genes within each subset of the Venn diagram reported in (c).

Proteomic analysis does not differentiate between different cellular mechanisms for protein release into the extracellular environment. We therefore defined a bioinformatic workflow for categorizing the differentially expressed proteins in the OA synoviocytes secretome following BC and CS treatments as proteins secreted via classical (through signal peptide) and non-classical (leaderless protein secretion) pathways using the SignalP and SecretomeP prediction algorithms, respectively (Figure 1b). Moreover, unconventional secretion of transmembrane proteins was also considered (Figure 1b). Of the proteins detected in the conditioned media of the OA synoviocytes and differentially expressed following BC and CS treatments, a high percentage (47%) are predicted to contain a signal peptide, whereas 10% of proteins are predicted to be secreted via the non-classical secretory pathway (Figure 2a).

Together, these predictions account for 57% of the proteins for which changes in the expression levels were detected in at least one treatment. In addition to classical and non-classical secretion, we also evaluated the percentage of transmembrane proteins (10%) that are known to be released from cell surface into the extracellular milieu via ectodomain shedding.

Interestingly, an enrichment analysis performed for the cellular component GO category (Figure 2b) revealed that a significant number of differentially expressed proteins belongs to exosomes (53%) and lysosomes (48%). Since there is growing evidence that proteins released via vesicles including exosomes are used by cells for intercellular communication, we investigated if exosome-related proteins identified by enrichment analysis were predicted to be secreted using the SecretomeP/Signal P prediction algorithms. Although 19 proteins were predicted to contain a signal peptide and seven were predicted to be secreted via non-classical secretion pathways, we found that for additional 16 proteins these algorithms did not predicted a potential release from cells (Figure 2c,d). This observation reflects the evolution of the term “secretome” that should also include proteins from various subcellular locations that may be released outside the cell through different mechanisms such as exosomes.

### 2.3. Comparative Analysis of the OA Synoviocytes Secretome Response to BC and CS Treatments

**Figure 3:**
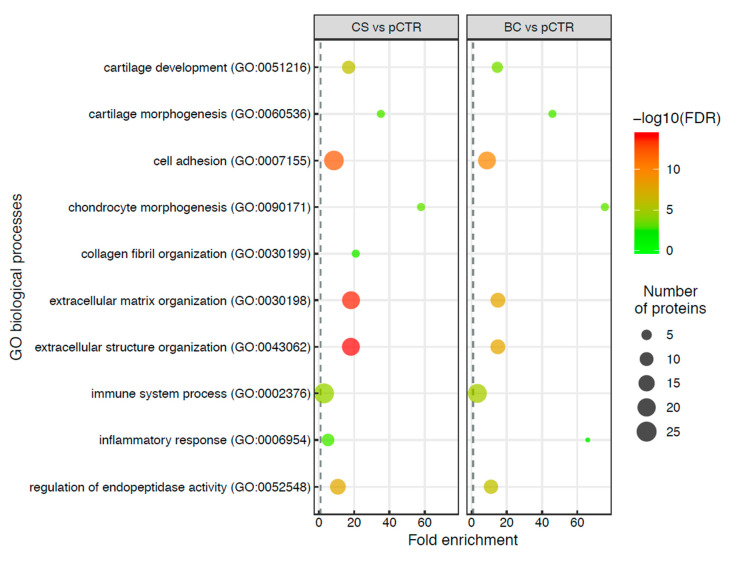
Dot plot of the top 10 over-represented biological processes of differentially expressed proteins in secretomes of CS-/BC-treated vs. pCTR synoviocytes. The dot size is proportional to the number of differentially expressed proteins associated with the process and the dot colour gradients indicated the significance of the enrichment (-log10(FDR corrected *p*-values)).

In addition to the cellular component enrichment analysis, we also investigated if there was a correlation of responses to different chondroitin treatments by evaluating the GO biological processes of differentially expressed proteins. We found that both treatments affected almost the same cellular processes, including the extracellular matrix (ECM) organization/ECM structure organization, cell adhesion, cartilage development, and morphogenesis (Figure 3).

Given the overlap in the biological processes and potential related signaling components affected by CS and BC treatments, at first, we evaluated the response of the common proteins identified in the two treatments. Indeed, we found a subset of proteins whose levels changed in the same directions under different treatments (i.e., up- or down-regulated). Among these, extracellular matrix proteins (e.g., Fibrillin-1, *FBN1*; Periostin, *POSTN*), enzymes (i.e., Serine protease *HTRA1*), as well as the Metalloproteinase inhibitor 2 (*TIMP2*) were commonly up-regulated upon BC and CS treatments. By contrast, other enzyme inhibitors such as Cystatin-A (*CYTA*) and several proteins involved in cell-cell adhesion (Desmocollin-1, *DSC1*; Desmoglein-1, *DSG1*; Desmoplakin, *DESP*; Junction plakoglobin, *PLAK*; Annexin A2, *ANXA2*) were down-regulated upon both CS and BC treatments.

**Figure 4:**
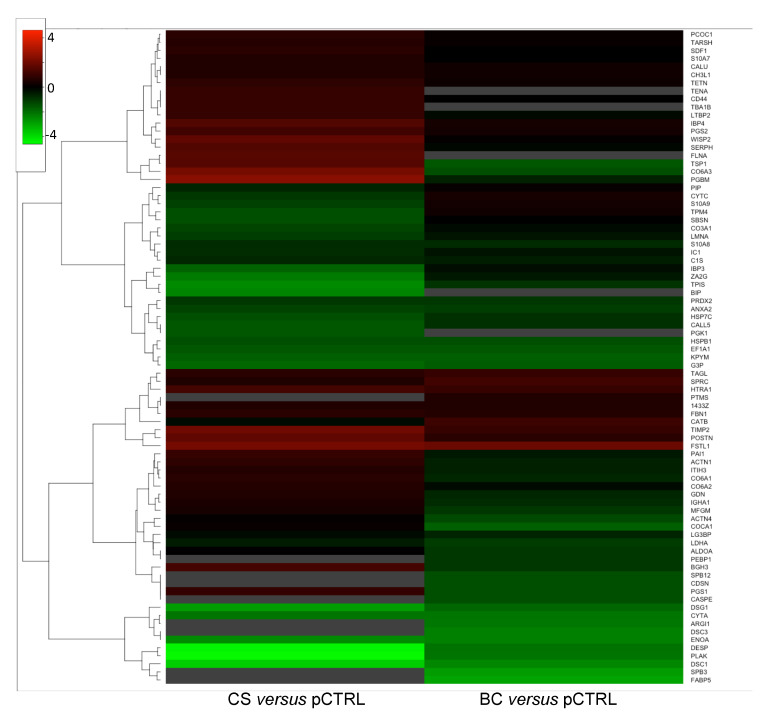
Heatmap representing the log2 fold-change values of differentially expressed proteins in CS-/BC-treated vs. pCTR synoviocytes secretomes. Down-regulated and up-regulated proteins are coloured in green and red, respectively. Missing expression values for undetected proteins in one out of the two conditions are reported in grey.

Despite this overall similar response to the different treatments, the comparative analysis of protein fold changes also revealed, for some differentially expressed proteins, a distinctive response of OA synoviocytes to BC and CS. These proteins, clearly clustered in the heatmap shown in Figure 4, include ECM proteins such as Collagen type VI chains (*CO6A1* and *CO6A3*) and Biglycan (*PGS1/BGN*), cell adhesion proteins such as the Transforming growth factor-beta-induced protein ig-h3 (*BGH3/TGFBI*) and the adhesive glycoprotein Thrombospondin-1 (*TSP1/THBS1*) that mediates cell-to-cell and cell-to-matrix interactions and the serine protease inhibitor Glia-derived nexin (*SERPINE2/GDN*). These protein changes were observed to be dependent on the different types of chondroitins. In particular, they were all up-regulated following CS treatment and, by contrast, down-regulated upon BC addition, suggesting the occurrence of both common and distinct regulation responses to chondroitin treatments.

**Figure 5:**
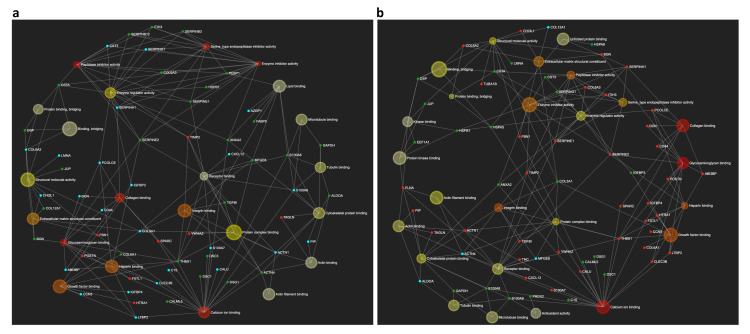
Bipartite view of the networks of enriched “molecular function” GO categories for the differentially expressed proteins in secretomes of (**a**) CS-treated vs. pCTR and (**b**) BC-treated vs. pCTR synoviocytes. The size of the node corresponds to the number of proteins included in the list and mapped on the specific term. The colour scale of the nodes is reported according to the *p*-value of the enriched category. The smaller nodes, corresponding to individual proteins/genes, are depicted in red (up-regulation) and green (down-regulation) based on their log 2 fold-change values. No change nodes are in light blue.

These findings were further supported by the enrichment network analysis performed on differentially expressed proteins following CS (Figure 5a) and BC (Figure 5b) stimulations. Indeed, a distinct response to treatments is observed by mapping identified proteins, along with their fold change levels, in association with corresponding significant GO molecular functions terms. The enrichment analysis also revealed that the most represented terms within the network were those of proteins involved in collagen and glycosaminoglycan binding, calcium ion binding, and growth factor binding, confirming that treatments affected similar biological processes by modulating both common and peculiar signaling events.

**Figure 6:**
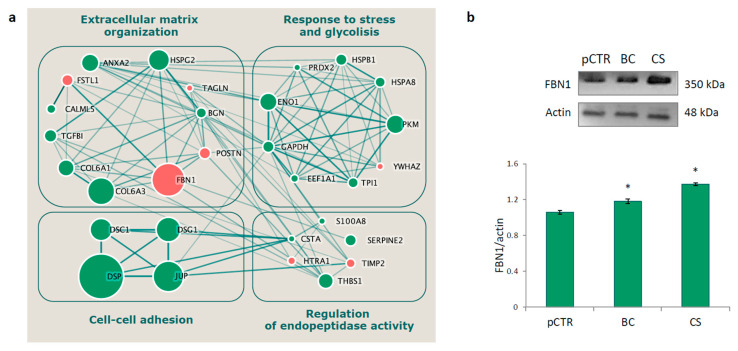
(**a**) Cytoscape network of enriched “biological processes” GO categories for proteins differentially expressed in both CS- and BC-treated with respect to pCTR synoviocytes secretomes identified by high-resolution LC-MS/MS. Up- and down-regulated proteins are reported as red and green nodes, respectively. Node size and edge thickness are related to number of peptides identified by MS and interaction confidence scores, respectively. Network clustering was performed according to the top enriched terms from the Cytoscape stringApp functional enrichment analysis (FDR-corrected *p*-value < 0.05) after redundancy filtering; (**b**) Validation of FBN1 up-regulation by Western blot analysis in CS- and BC-treated OA synoviocytes. β-Actin was used as the loading control. Relative changes of treated samples versus pCTR are given as mean + SD of three independent analyses, * *p* < 0.05.

An enrichment analysis was also performed to identify significant GO biological processes associated to differentially expressed proteins in both CS- and BC-treated secretomes (Figure 6a). The enrichment analysis revealed that several differentially expressed proteins were involved in cell-cell adhesion (FDR: 1.56 × 10^−7^), regulation of endopeptidase activity (FDR: 5.78 × 10^−5^) and response to stress/glycolysis (FDR: 7.43 × 10^-6^). In addition, several proteins are implicated in extracellular matrix organization (FDR: 6.27 × 10^−10^), including Fibrillin-1 the up-regulated protein identified with the highest number of peptides within this cluster (Figure 6a). In line with MS data, Western blot analysis of FBN1 performed on BC- and CS-treated OA synoviocytes lysates showed a similar increase in protein expression following both BC and CS treatments (Figure 6b). The list of differentially expressed proteins in both CS- and BC-treated secretomes was then subjected to STRING (v. 11.0) analysis to reveal enriched reactome pathways related to treatments. This analysis further confirmed that ECM organization and degradation were the top enriched pathway terms within the String functional interaction network (Supplementary Figure S1).

### 2.4. Comparative Analysis of Differentially Regulated Proteins in OA Synoviocytes and Chondrocytes Secretomes in Response to Different Chodroitin Treatments

To investigate differences between OA chondrocytes (hOAC) and OA synoviocytes (hOAS) responses to chondroitin sulphate treatments, we compared our results with those previously reported in secretome studies using quantitative MS approaches [21,24]. In these studies, the effects on secretomes were evaluated on human OA chondrocyte following treatments with bovine and porcine chondroitins. Overlap of differentially expressed proteins were combined and visualized using an UpsetR plot (Supplementary Figure S2a) and a chord diagram graph (Supplementary Figure S2b). As expected, each treatment mostly affects unique responses in hOAC or hOAS (Supplementary Figures S2 and S3a). Nevertheless, 17 proteins were found to be commonly differentially expressed in hOAC and hOAS under specific chondroitin treatments (Supplementary Figures S2 and S3b). Of these, the three proteins encoded by *SERPINE2*, *THBS1*, and *TGFBI* genes were identified in all datasets, strongly suggesting for some differentially expressed proteins a modulation by chondroitin treatments independent of the specific cell type.

### 2.5. Targeted Cytokines Profiling by Multiplex Immunoassay

Based on the pivotal role of inflammation in OA development and/or progression, there is a growing interest in determining biological mediators responsible of catabolic and anabolic effects occurring in response to the inflammatory process. Despite the fact that roles played by the plethora of these mediators have not been fully clarified yet, a crucial connection with several cytokines is widely recognized.

**Figure 7:**
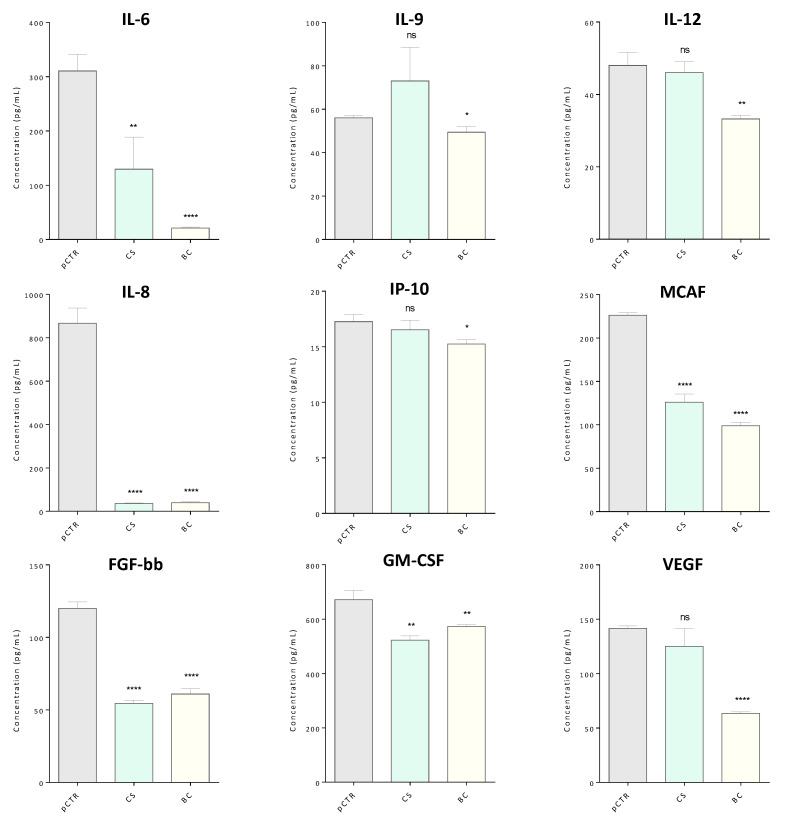
Expression levels of significantly differentially modulated cytokines in CS-/BC-treated with respect to pCTR synoviocytes secretomes. Measured concentrations are referred to CM collected from 10 × 10^4^ cells for all conditions. CM were simultaneously screened for determining the cytokines concentration by interpolation on standard curves. All measurements were performed in triplicate. Data are reported as means ± SD. (* *p* ≤ 0.05; ** *p* ≤ 0.01; *** *p* ≤ 0.001; **** *p* ≤ 0.0001).

High-throughput -omics strategies provide an integrated view of biological regulatory networks and pathways. However, the high dynamic range of biological systems makes the study of complex matrices especially challenging for the detection of low-abundance proteins. In order to integrate our secretome survey by the MS approach, we performed a multiplex immunoassay for the simultaneous measurement of 27 low-abundance analytes (e.g., cytokines, chemokines, growth factors) within the OA synoviocytes secretome following BC and CS treatments. A small subset of analytes was found to be significantly modulated in treated compared to untreated synoviocytes (Figure 7).

In particular, we found that the BC treatment decreased the levels of nine biological mediators out of the 27 assayed, namely IL-6, IL-8, IL-9, IL-12, FGF-bb, GM-CSF, IP-10, MCAF, and VEGF. The most significant differences were observed for IL-6, IL-8, FGF-bb, VEGF and MCAF (*p* ≤ 0.0001). For IL-6, IL-8, FGF-bb, and MCAF, significant lower levels were also observed upon CS treatment. This last treatment also induced a decrease of GM-CSF, while did not affect the expression levels of IL-9, IL-12, IP-10 and VEGF. In addition, no significant differences were detected for both treatments in the expression levels of (IL)-1β, IL-1ra, IL-2, IL-4, IL-5, IL-10, IL-13, IL-15, IL-17A, Eotaxin, G-CSF, IFNγ, MIP-1α, MIP-1β, RANTES, TNFα, PDGF-BB (Supplementary Table S2), while no detectable levels were revealed for IL-7 in the analyzed samples.

## 3. Discussion

In recent years, global proteomic studies based on mass spectrometry approaches have been widely applied to investigate the pathophysiology of articular cartilage (extensively reviewed in [26]). To date, most studies have been focused on proteins directly identified in the secretome of chondrocyte cultures [20,22,24,27,28,29,30,31,32,33,34,35]. In addition, proteomic analyses were also performed on cartilage tissues and cartilage explants [26]. Nevertheless, most of proteome and secretome research targets chondrocytes, also to study the effects of different chondroitin treatments (e.g., bovine CS, porcine CS) and formulations in OA models [21,22,23,24,36]. Secretome studies by high-resolution mass spectrometry on primary human synoviocytes, the main cellular components of the synovium, are lacking. Indeed, to date, a phosphoproteomic analysis of synoviocytes has only been reported by Tang and co-workers [37]. In addition, extensive proteomic characterizations have been performed so far on OA synovial fluids [38,39,40,41,42,43,44]. Several studies also focused on the characterization of proteomic changes occurring in synovial fluids at different OA stages (i.e., traumatic arthritis, early-stage and late-stage) [41,43,45,46]. This is probably due to difficulties in setting up pathological synoviocytes primary cultures. Synoviocytes, within the joint, produce glycoproteins that are secreted in the synovial fluid, essential for joint lubrication. Upon the onset of inflammatory diseases, including OA, a high prevalence of synovial inflammation occurs, leading to significant changes, such as the synovial lining layer and production of inflammatory cytokines [41,47]. It is increasingly recognized that the acquisition of these abnormal molecular and morphological features may contribute to OA onset and structural progression [41,47].

In the current study, a label-free quantitative high-resolution mass spectrometry-based proteomic approach has been complemented with a multiplex protein array approach to investigate the extracellular responses of OA synoviocytes to treatments with a marine CS or with unsulfated biotechnological chondroitin, for which literature data are still missing. This strategy allowed the identification of candidate proteins that potentially correlate with treatment outcome, several of which have been previously correlated to healthy and OA chondrocyte secretome [26]. These proteins can be mainly classified as ECM proteins, enzymes and enzymatic inhibitors, cytokines and growth factors. As expected, we found that many of the differentially expressed proteins upon both CS and BC treatments comprises proteins released through various mechanisms including classical and non-classical secretory pathways as well as exosome-mediated secretion and membrane shedding. These findings support the hypothesis that these proteins released from synoviocytes in response to chondroitin stimulation may play a role in communication with neighboring cells by acting in an autocrine or paracrine manner. One of the major focus of our study was to investigate similarities and differences between CS and BC chondroitin treatments. An overall agreement in the trend of responses was observed for several up- and down-regulated proteins involved in ECM structure organization, cell adhesion and cartilage biological processes.

It is challenging to compare our data with previously published data sets, mainly performed on chondrocytes secretomes, because of the differences in cell types (e.g., chondrocytes, cartilage tissues, synovial fluids) and in quantitative mass spectrometry approaches [26]. In addition, previous literature also highlights a huge variability of the effects on chondrocytes upon different treatments (e.g., different types of chondroitin sulfate compounds), formulations (CS alone or combined with glucosamine sulfate/hydrochloride) and stimulations (e.g., IL-1 β) [26]. However, consistent with our findings are previous studies reporting the modulation of several secreted proteins, mainly ECM proteins and growth factors, following chondrocyte treatment with CS isolated from different animal sources, both alone or combined with other agents [21,22,23,24].

A comparison with an updated catalogue of proteins identified by quantitative mass-spectrometry-based studies [26] revealed a significant overlap of our subset of differentially expressed proteins with previously identified proteins secreted by human chondrocytes and cartilage (Supplementary Figure S4). Interestingly, we found that some proteins (i.e., *SERPINE2*, *THBS1*, and *TGFBI*) were differentially regulated in both OA synoviocytes and chondrocytes secretomes in response to different chondroitin treatments (i.e., bovin, porcine, marine and BC) suggesting, for some differentially expressed proteins, a modulation by chondroitin treatments independent from the specific cell type. In addition, several proteins reported earlier in OA synovial fluid were also identified in our study supporting the advantages of complementing synovial fluids characterization with secretome profiles of synoviocytes [40]. Several differentially expressed proteins show a similar regulation response following CS and BC treatments with respect to pCTR. Among proteins up-regulated in both conditions, we identified some extracellular matrix proteins that have been previously implicated in osteoarthritis pathogenesis. Among these, elevated expression levels Follistatin-related protein 1 (*FSTL1*) [48] and Periostin (*POSTN*) [49] were previously correlated to OA progression and severity. In addition, increased expression levels of Fibrillin-1 (*FBN1*), the major constituent of tissue elastic microfibers and previously identified also in human osteoarthritis synovial fluids [40], were identified by MS secretome analysis and confirmed by Western blot. Similarly, increased expression levels of the Serine protease *HTRA1* have been found in OA cartilage [50,51]. This protease was found to be up-regulated by up to eight-fold in OA cartilage as compared to normal cartilage [51,52]. Accordingly, also a sevenfold increase of *HTRA1* mRNA levels has been reported by Hu et al. [53]. Although for this and other ECM proteins an up-regulation is still observed following our treatments, we found fold change levels much lower than those previously correlated with OA, likely in response to CS and BC treatments. Notably, in our study, the Metalloproteinase inhibitor 2 (*TIMP2*) is up-regulated upon both CS and BC treatments. In fact, metalloproteases (MMPs) are well-known to be involved in cartilage matrix degradation in OA [50]. By inhibiting the ECM metalloproteinases, TIMPs exert a cartilage protective role in OA. A protective role on cartilage induced by both treatments can be also hypothesized based on the down-regulation in CS- and BC-treated synoviocyte secretomes of other proteins involved in tissue breakdown such as Annexin A2 (*ANXA2*). Indeed, high levels of *ANXA2* have been measured in several immune-mediated diseases and in synovial tissues of patients with rheumatoid arthritis and osteoarthritis, causing cartilage destruction by promoting migration and invasion of fibroblast-like synoviocytes into cartilage [54,55]. In addition, among commonly down-regulated proteins we identified the Protein S100-A8 (*S10A8*), previously reported to be secreted by chondrocytes and to play a role in cartilage degradation in inflammatory arthritis by acting as an unconventional pro-inflammatory cytokine [56].

Despite similarities among CS and BC treatments, for several up- and down-regulated proteins in OA, an opposite or different regulation trend was observed, suggesting their involvement in the response to drugs. Among these proteins, we identified the small proteoglycans Biglycan (*PGS1/BGN*) and Decorin (*PGS2/DCN*). High levels of soluble forms of *BGN* and *DCN* were found in synovial fluid of OA or rheumatoid arthritis patients. In particular, a role of *BGN* as a mediator of OA cartilage degradation through TLR4 signalling has been hypothesized, thus indicating the involvement of this protein in the loss of cartilage [57]. In our study, *BNG* was up-regulated upon CS treatment (fold change = 1.9) while a decrease was observed in BC-treated synoviocytes (fold change = 0.4). A similar response was observed for the large heparan sulfate proteoglycan Perlecan (Basement membrane-specific heparan sulfate proteoglycan core protein, *PGBM*), that was found strongly up-regulated in CS-treated synoviocytes CM (fold change = 5.6) and slightly down-regulated in BC-treated sample (fold change = 0.6). Synovial Perlecan has been shown to play a key role in osteophyte formation in OA. Up-regulated levels of Perlecan in response to TGF-β have been also detected in the synovium from OA patients [58]. Interestingly, Kaneko and co-workers demonstrated that synovial Perlecan deficiency inhibited osteophyte formation suggesting that dysregulation of proteoglycan metabolism plays an important role in the pathology of OA [59]. Two additional proteins (i.e., transforming growth factor-beta-induced protein ig-h3 and thrombospondin-1), previously reported to be highly secreted by OA chondrocytes, were identified among proteins showing a different response to our chondroitin treatments, thus suggesting the occurrence of both similar and different effects on molecular mechanisms activated by drugs.

This observation is further supported by our targeted cytokines profiling performed by multiplexed immunoassay demonstrating that both treatments significantly decreased the levels of pro-inflammatory cytokines (IL-6), chemokines (IL-8 and MCAF), and growth factors (FGF-basic and GM-CSF). Furthermore, BC treatment alone selectively modulated other secreted mediators (i.e., VEGF, IL-9, IL-12, and IP-10). Most of these factors have a well-known implication in OA pathophysiology. Indeed, IL-6 and IL-12 are closely related to the OA inflammatory process and their increase is also associated to the progressive loss of joint function [60,61]. Similarly, IL-9 enhances the production of several pathogenic mediators amplifying the OA-related inflammatory response [62] and MCAF is also known to be related to the pro-inflammatory cascade [63]. Moreover, recent studies have reported a correlation between VEGF production and OA severity [61]. GM-CSF is responsible of inflammation progress and it has been recently correlated to OA pain [64]. Since synoviocytes are fibroblast-like cells and their massive growth is correlated to invasiveness of the synovial tissue, the observed decrease of fibroblast growth factor (FGF-bb), a widely known pro-proliferative factor for fibroblasts, could be considered a positive outcome of both treatments [65]. Many pharmacoproteomic studies on the effects of treatments with chondroitin sulfate from diverse animal sources focus on chondrocytes as model system. Our findings improve the current knowledge of the biochemical effects driven by novel extractive sulfated and biotechnological unsulfated chondroitin-based drugs on primary synoviocytes and further demonstrate the usefulness of analyzing secretomes of human osteoarthritic synoviocytes to investigate the molecular effects of SYSADOA therapies. Future perspectives of this study will include the evaluation of the effects of chondroitins under different treatment conditions as well as at intracellular level to gain insight into molecular pathways involved in different responses to treatments.

In vitro investigations, even with limited direct clinical impacts, are necessary and preliminary to plan in vivo studies on a large cohort of patients for validating the relevance of identified molecules in pathways associated to osteoarthritis pathogenesis. Only these latter studies will then be able to provide clear and direct information on the effects of chondroitin treatments in OA pathogenesis.

## 4. Materials and Methods

### 4.1. Preparation of CS and BC Based Gels

Chondroitin sulfate (95 ± 5% purity) extracted from shark cartilage was provided by IBSA (condrosulf^®^400 lot.: 151001) with a very low endotoxin content (0.1 EU/mg). Biotechnological chondroitin (95 ± 5% purity) was produced in our laboratories and purified to pharma grade as previously reported [66,67] and its endotoxin content was evaluated through Limulus test (EU/mg < 0.05). CS and BC were dissolved in 20 mL Phosphate-Buffered Saline (PBS, pH 7.2, Lonza, Milan, Italy) at a concentration of 16 mg/mL. pH and osmolality were measured in order to perform experiments under physiological conditions (i.e., pH 7.0 ± 0.1 and osmolality 300 mOsm). The solutions were sterilized by autoclave (1 bar, 121 °C, 20 min). Finally, glycosaminoglycan-based formulations were diluted in the culture medium (with or without fetal bovine serum Gibco FBS, Fisher Scientific Italia, Milan, Italy) at a 3.2 mg/mL final concentration.

### 4.2. In Vitro Cell Cultures and Glycosaminoglycan Treatments

Synoviocytes cells were isolated as previously reported [16,68] from three women OA patients (58, 60, and 63 years old) undergoing the surgical procedure of knee joint replacement at Orthopedics and Traumatology Department of University Federico II of Naples. The patients gave informed consent and the procedures were approved by Internal Ethical Committee. Specifically, synoviocytes were obtained by isolating primary cells from the synovial fluids obtained during surgical procedures. Cell phenotype was assessed by FACS characterization of specific biomarkers as previously described [16,68], revealing that the population of cells isolated (at first passage of culture) was mainly composed of type B synoviocytes. For the synoviocytes treatments, we followed a slightly modified cellular starvation protocol described by Calamia and collaborators [15]. In particular, 10 × 10^4^ cells/cm^2^ into a 75 cm^2^ flask were seeded and grown to 80% confluence. Then, following the extensive washing of cells with PBS, medium containing 0.5% FBS was added. After 24 h, cells were further washed five times (10 mL each) with PBS and cultured in serum-free medium with or without CS (3.2 mg/mL) and BC (3.2 mg/mL) for 48 h. CS and BC concentrations used in this study were optimized in accordance with previous investigation [69]. At the end of incubation, media were centrifuged (1500 rpm, 7 min) and supernatants stored at −80 °C.

### 4.3. Targeted Quantitative Analysis of Secreted Cytokines by Bio-Plex Assay

The targeted quantitative analysis of secreted cytokines and chemokines in culture media (CM) was performed by using the Bio-Plex multiplex system (Bio-Rad, Milan, Italy) based on xMAP technology [70,71]. Media collection was performed following a 48-h treatment with CS and BC as described in Section 4.2. The 48-h treatment time was selected according to previously optimized protocols [16,68]. All steps were performed according to manufacturer’s instructions. The concentration of the following analytes were simultaneously determined within CM of untreated (pCTR) and CS- or BC-treated synoviocytes: interleukin (IL)-1β, IL-1ra, IL-2, IL-4, IL-5, IL-6, IL-7, IL-8, IL-9, IL-10, IL-12 (p70), IL-13, IL-15, IL-17A, IP10, Eotaxin, Granulocyte-colony stimulating factor (G-CSF), Granulocyte macrophage colony stimulating factor (GM-CSF), Interferon (IFN)γ, Monocyte chemoattractant protein 1 (MCAF/MCP-1), Macrophage inflammatory protein 1-alpha and beta (MIP-1α and MIP-1β), RANTES, Tumor necrosis factor alpha (TNFα), Platelet-derived growth factor-BB (PDGF-BB), Vascular endothelial growth factor (VEGF), Basic fibroblast growth factor (FGF-basic). Data were acquired using a Bio-Plex MAGPIX Multiplex Reader system (Bio-Rad).

Data were expressed as mean ± SD. A two-tailed t-test was used to assess the significance between the CS- and BC-treated synoviocytes CM samples with respect to pCTR by using the GraphPad Prism software v 5.0 (La Jolla, CA, USA). Expression data were also imported into the excel software for further analyses.

### 4.4. Sample Preparation for High Resolution nanoLC-Tandem Mass Spectrometry Analyses

CM collected from untreated (pCTR) and CS- and BC-treated osteoarthritic human synoviocytes were concentrated to a final volume of 500 µL with Amicon ultrafiltration units (MWCO 3kDa, Millipore, Billerca, MA, USA) and subjected to trichloroacetic acid (TCA) precipitation [72]. Protein pellets were air dried and then resuspended in 50 µL of triethylammonium bicarbonate (TEAB). Protein concentration was determined by Quantum Micro Protein Assay Kit- BCA Total Protein Assay Kit for dilute samples (Euroclone S.p.A., Milan, Italy). Equal amounts of proteins (10 µg) were reduced, alkylated and digested as reported elsewhere [73]. Following tryptic digestions, samples were centrifuged at 10,000× *g* for 15 min and supernatants were dried under vacuum in a SpeedVac Vacuum (Savant Instruments, Holbrook, NY, USA). Samples were then resuspended in 25 μL of H_2_O/TFA 2% and centrifuged at 10,000× *g* for 15 min. Aliquots of the samples (5 μL) were analysed in triplicate on a Q-Exactive Orbitrap mass spectrometer equipped with an EASY-Spray nano-electrospray ion source (Thermo Fisher Scientific, Bremen, Germany) and coupled to a Dionex UltiMate 3000RSLC nano system (Thermo Fisher Scientific) [74,75].

### 4.5. Database Searching and Label Free Quantitation

The acquired raw files were analysed with the Proteome Discoverer 2.1 software (Thermo Fisher Scientific). For label-free quantification by spectral counts, each raw file was run separately in batch mode using a standard Sequest HT-Target Decoy PSM validator workflow. Briefly, the HCD MS/MS spectra were searched against the Homo sapiens Uniprot_sprot database (release 2019_11, 20,380 entries). The parameter settings were as follows: mass tolerance of 10 ppm for precursors and 0.02 Da for ion fragments; two missed cleavages allowed; trypsin as enzyme specificity. Carbamidomethylation of cysteine (+57.021 Da) was set as static modification while oxidation of methionine (+15.995 Da), N-terminal acetylation (+42.011 Da) and phosphorylation of serine, threonine and tyrosine (+79.966 Da) were considered as dynamic modifications. A minimum number of six amino acids were required for peptide identification. Processing results are then used for protein identification by using a single Consensus workflow with the “Merge Mode” parameter in the MSF files node set to “Do Not Merge”. With this setting, the number of unique peptides and PSMs are obtained for each condition. False discovery rates (FDRs) for peptide spectral matches (PSMs) were calculated and filtered using the Target Decoy PSM Validator node in Proteome Discoverer on the basis of dynamic score-based thresholds. Target Decoy PSM Validator settings were: Maximum Delta Cn 0.05, a strict target FDR of 0.01, a relaxed target FDR of 0.05 and validation based on q-value. Proteins with a q-value of <0.01 were classified as high confidence identifications and proteins with a q-value of 0.01–0.05 were classified as medium confidence identifications. PSM values obtained for each protein are then exported to Excel for manual calculation of spectral counts-based ratios. Only proteins identified with high/medium confidence in two out of three replicates and with more than one peptide in at least one out of three conditions were retained. Proteins with fold change ratios ≥1.5 and ≤0.66 in CS-treated and BC-treated synoviocytes with respect to untreated control cells were considered as differentially expressed.

### 4.6. Bioinformatic Analyses

Functional enrichment based on cellular component gene ontology (GO) category was performed by the FunRich open access software (http://funrich.org/index.html). The “molecular function” enrichment network of differentially expressed proteins was constructed by using the Network Analyst platform (https://www.networkanalyst.ca) [71]. The “biological process” enrichment network of differentially expressed proteins was constructed using the STRING database implemented in the StringApp plug-in for Cytoscape software 3.7.2 [76]. Briefly, StringApp builds a network of the input proteins and compares its connectivity (number of interactions) to the connectivity of other networks of similar size generated with random sets of proteins. To test whether GO terms were enriched in the input protein list, enrichment analyses were conducted with the StringApp using the human GO dataset as a reference and a cutoff value of FDR < 0.05. The enrichment analysis of pathways for differentially expressed proteins in both CS- and BC-treated secretomes was performed by using the STRING (v. 11.0) resource available online at https://string-db.org/ against the Reactome curated pathways database.

Analyses of secretion pathways for differentially expressed proteins were performed according to SecretomeP 2.0 Server (http://www.cbs.dtu.dk/services/SecretomeP/). If the neural network exceeded or was equal to a value of 0.5 (NN-score ≥ 0.50) and no signal peptide is predicted, proteins were considered to be potentially secreted via non-classical pathways. Those proteins with a predicted N-terminal signal sequence were confirmed using SignalP 5.0, available at http://www.cbs.dtu.dk/services/SignalP/and were considered to be secreted via a classical pathway (endoplasmic reticulum/Golgi-dependent pathway). Finally, TMHMM 2.0 server (http://www.cbs.dtu.dk/services/TMHMM/) was used to predict the existence of α-helical transmembrane domains. Plots of selected over-represented biological processes GO terms for differentially expressed proteins in response to CS and BC treatments were generated from a homemade R script and the ggplot2 R package of the RStudio v 1.2.1335 environment for R (http://www.R-project.org). The list of representative enriched GO terms was obtained using Panther [77] and REVIGO [78] tools. The Enhanced Heat Map (heatmap.2) function from the gplots R package was used to generate the heat maps of differentially expressed proteins. The UpSetR plot for comparative analysis of differentially regulated proteins in OA synoviocytes and chondrocytes secretomes in response to different chondroitin treatments was generated by using the UpSetR Shiny App available on-line at https://gehlenborglab.shinyapps.io/upsetr/ [79]. Datasets of differentially expressed proteins from [21,24] were combined to visualize set intersections in a matrix layout. The chord diagram associated to the UpSetR plot was constructed by using the Network Analyst platform (https://www.networkanalyst.ca) [71].

### 4.7. Western Blot Analysis

In order to evaluate the expression levels of Fibrillin-1, a Western blotting analysis was performed on untreated and BC- or CS-treated cell extracts harvested with trypsin/EDTA 0.2 mg/mL and lysed by a Radio-Immunoprecipitation Assay (RIPA buffer 1×; Cell Signaling Technology). The protein concentration for each sample was assayed through Bradford method. We used 8% (stacking gel) and 12% (separating gel) SDS-PAGE to electrophoretically separate intracellular proteins (10 μg/lane) before transfer onto nitrocellulose membrane (GE, Amersham, UK). After the transfer, the membrane was blocked by 5% non-fat milk in Tris-buffered saline and 0.05% Tween-20 (TTBS) for 15 min. Then, primary antibody against Fibrillin-1 (Fibrillin-1 Monoclonal Antibody 11C1.3; Thermo Fisher Scientific) was diluted 1:100 and incubated overnight at 4 °C. Afterwards, the membrane was washed using TTBS and an anti-mouse horseradish peroxidase-conjugated secondary antibody (Santa Cruz Biotechnology, Dallas, TX, USA), diluted 1:5000 was incubated for 2 h at room temperature. Anti-β-Actin antibody used at 1:1000 dilution was used as the loading control. Finally, chemoluminescent signals were acquired by ECL system (Millipore) and the semi-quantitative analyses of protein expression was carried out by ImageJ program. Relative changes of treated samples versus pCTR are given as mean + SD of three independent analyses.

## 5. Conclusions

It is well accepted that OA is a chronic and multifactorial disease affecting human joints. Secretome investigations using proteomic approaches proved to be promising to unravel molecular mechanisms underlying the disease and pharmacological effects of SYSADOA. In this study, the integration of a targeted multiplexed protein array analysis and of an untargeted mass spectrometry-based approach enables an unbiased profiling of the protein expression changes in response to unsulfated and marine sulfated chondroitins. Despite the absence of sulphate groups in the biotechnological chondroitin, often reported of key importance in molecular recognition and bioactivity, we found similar protein changes at extracellular level driven by CS and BC. In addition, results of this study improve knowledge on CS effects by gaining insights on molecular determinants affected by a more sulphated and larger biopolymer (Mw of marine CS > Mw of terrestrial CS). Our study also paves the way for future research aimed to unravel potential efficacy of unsulfated microbially derived chondroitin in counteracting OA-related pathological processes.

## Figures and Tables

**Table 1:** Proteins differentially expressed (0.6 ≥ FC ≥ 1.5) in both CS- and BC-treated with respect to pCTR synoviocytes secretomes identified by high-resolution LC-MS/MS.

Accession	Gene	Description	CS vs. pCTR	BC vs. pCTR
P63104	*1433Z*	14-3-3 protein zeta/delta	1.5	1.5
P35555	*FBN1*	Fibrillin-1	1.7	1.5
Q12841	*FSTL1*	Follistatin-related protein 1	4.3	3.8
P16035	*TIMP2*	Metalloproteinase inhibitor 2	4.0	2.0
Q15063	*POSTN*	Periostin	3.3	1.7
Q92743	*HTRA1*	Serine protease HTRA1	2.3	2.0
Q01995	*TAGL*	transgelin	1.7	2.0
P06733	*ENOA*	alpha-enolase	0.2	0.2
Q9NZT1	*CALL5*	Calmodulin-like protein 5	0.3	0.6
P01040	*CYTA*	Cystatin-A	0.2	0.2
Q08554	*DSC1*	Desmocollin-1	0.1	0.2
Q02413	*DSG1*	Desmoglein-1	0.1	0.3
P15924	*DESP*	Desmoplakin	0.0	0.2
P68104	*EF1A1*	Elongation factor 1-alpha 1	0.3	0.3
P04406	*G3P*	glyceraldehyde-3-phosphate dehydrogenase	0.3	0.3
P11142	*HSP7C*	Heat shock cognate 71 kDa protein	0.4	0.6
P04792	*HSPB1*	Heat shock protein beta-1	0.4	0.4
P07355	*ANXA2*	Isoform 2 of Annexin A2	0.4	0.5
P14923	*PLAK*	Junction plakoglobin	0.0	0.2
P32119	*PRDX2*	Peroxiredoxin-2	0.5	0.5
P05109	*S10A8*	Protein S100-A8	0.6	0.6
P14618	*KPYM*	Pyruvate kinase PKM	0.3	0.3
P60174	*TPIS*	Triosephosphate isomerase	0.2	0.5
P21810	*PGS1*	biglycan	1.9	0.4
P12109	*CO6A1*	Collagen alpha-1(VI) chain	1.6	0.6
P12111	*CO6A3*	Collagen alpha-3(VI) chain	4.4	0.4
P07093	*GDN*	Isoform 3 of Glia-derived nexin	1.5	0.6
P07996	*TSP1*	thrombospondin-1	2.9	0.3
Q15582	*BGH3*	Transforming growth factor-beta-induced protein ig-h3	2.3	0.5
P98160	*PGBM*	Basement membrane-specific heparan sulfate proteoglycan core protein	5.6	0.6

**Table 2:** Proteins differentially regulated (0.6 ≥ FC ≥ 1.5) in CS- or BC-treated with respect to pCTR synoviocytes secretomes identified by high-resolution LC-MS/MS. N/D, not detected.

Accession	Gene Name	Description	CS vs. pCTR	BC vs. pCTR
P16070	*CD44*	CD44 antigen	2.0	1.0
P36222	*CH3L1*	Chitinase-3-like protein 1	1.5	1.3
P12110	*CO6A2*	Collagen alpha-2(VI) chain	1.6	0.9
P07585	*PGS2*	decorin	2.3	1.3
P21333	*FLNA*	Filamin-A	3.0	N/D
P22692	*IBP4*	insulin-like growth factor-binding protein 4	2.8	1.3
Q06033	*ITIH3*	Inter-alpha-trypsin inhibitor heavy chain H3	1.6	0.7
O43852	*CALU*	Isoform 3 of Calumenin	1.5	1.3
P12814	*ACTN1*	Isoform 4 of Alpha-actinin-1	1.8	0.7
P48061	*SDF1*	Isoform Delta of Stromal cell-derived factor 1	1.7	1.0
Q14767	*LTBP2*	Latent-transforming growth factor beta-binding protein 2	1.9	0.9
P05121	*PAI1*	Plasminogen activator inhibitor 1	2.0	0.8
Q15113	*PCOC1*	Procollagen C-endopeptidase enhancer 1	1.7	1.1
P31151	*S10A7*	Protein S100-A7	1.5	1.0
P50454	*SERPH*	Serpin H1	2.8	0.9
Q7Z7G0	*TARSH*	Target of Nesh-SH3	1.6	1.1
P24821	*TENA*	Tenascin	2.0	N/D
P05452	*TETN*	Tetranectin	1.7	1.2
P68363	*TBA1B*	Tubulin alpha-1B chain	2.0	N/D
O76076	*WISP2*	WNT1-inducible-signaling pathway protein 2	3.3	1.0
P11021	*BIP*	78 kDa glucose-regulated protein	0.2	N/D
P02461	*CO3A1*	Collagen alpha-1(III) chain	0.4	0.9
P09871	*C1S*	Complement C1s subcomponent	0.6	0.7
P01034	*CYTC*	Cystatin-C	0.5	1.3
P17936	*IBP3*	Isoform 2 of Insulin-like growth factor-binding protein 3	0.3	0.8
P05155	*IC1*	Isoform 3 of Plasma protease C1 inhibitor	0.6	0.8
P00558	*PGK1*	phosphoglycerate kinase 1	0.3	N/D
P02545	*LMNA*	Prelamin-A/C	0.5	0.8
P12273	*PIP*	Prolactin-inducible protein	0.6	1.1
P06702	*S10A9*	Protein S100-A9	0.5	1.3
Q6UWP8	*SBSN*	Suprabasin	0.4	1.0
P07226	*TPM4*	Tropomyosin alpha-4 chain	0.4	1.3
P25311	*ZA2G*	Zinc-alpha-2-glycoprotein	0.2	0.8
O43707	*ACTN4*	Alpha-actinin-4	1.1	0.4
P05089	*ARGI1*	Arginase-1	N/D	0.2
P31944	*CASPE*	Caspase-14	N/D	0.4
Q99715	*COCA1*	Collagen alpha-1(XII) chain	1.1	0.3
Q15517	*CDSN*	corneodesmosin	N/D	0.4
Q14574	*DSC3*	Desmocollin-3	N/D	0.2
Q01469	*FABP5*	Fatty acid-binding protein, epidermal	N/D	0.1
Q08380	*LG3BP*	Galectin-3-binding protein	0.9	0.6
P01876	*IGHA1*	Ig alpha-1 chain C region	1.4	0.6
P04075	*ALDOA*	Isoform 2 of Fructose-bisphosphate aldolase A	1.0	0.5
Q96P63	*SPB12*	Isoform 2 of Serpin B12	N/D	0.4
P00338	*LDHA*	Isoform 3 of L-lactate dehydrogenase A chain	0.7	0.5
Q08431	*MFGM*	Lactadherin	1.3	0.5
P30086	*PEBP1*	phosphatidylethanolamine-binding protein 1	N/D	0.5
P29508	*SPB3*	Serpin B3	N/D	0.1
P07858	*CATB*	Cathepsin B	0.9	2.1
P20962	*PTMS*	Parathymosin	N/D	1.5
P09486	*SPRC*	Sparc	1.5	2.3

## Supplementary Materials

Supplementary Materials can be found at https://www.mdpi.com/1422-0067/21/11/3746/s1. Figure S1. (a) STRING network of enriched “Reactome pathways” terms (b) for differentially expressed proteins in CS- and BC-treated with respect to pCTR synoviocytes secretomes identified by high-resolution LC-MS/MS. Node colors are related to enriched Reactome pathway terms. Nodes not included in the top enriched pathways are reported in grey. Edges represent protein-protein associations according to string color code. Figure S2. (a) UpsetR chart showing the overlap of differentially expressed proteins identified by high-resolution LC-MS/M following treatment of human OA synoviocytes (hOAS) with marine (mCS, green bar) and biotechnological (BC, light blue bar) chondroitins with differentially expressed proteins following treatment of human OA chondrocytes (hOAC) with bovine (bCS, yellow bar) and porcine (pCS, orange bar) chondroitins [21,24]. Details of proteins within each subset are reported in Figure S3 (a-b). Numbers of proteins shared between different data sets are indicated in the top bar chart and the specific treatments are indicated with solid dots below the bar chart. Total identifications for each dataset are indicated on the left as set size bars. (b) Chord diagram showing an overview of the overlap of differentially expressed proteins within different treatments. Segments of the arcs represent different treatments while the chords connecting them represent proteins. Figure S3. (a) Uniquely differentially expressed proteins in hOAC or hOAS following specific chondroitin treatments. (b) Commonly differentially expressed proteins in hOAC and hOAS under specific chondroitin treatments. Figure S4. Venn diagram showing the overlapping between dataset of differentially modulated proteins in CS-/BC-treated synoviocytes secretomes with the updated catalogue of proteins identified on human chondrocytes and cartilage secretomes by quantitative mass-spectrometry-based studies reviewed by Sanchez et. al. [26]. A comparison of fold changes ratios in CS-/BC-treated with respect to pCTR synoviocytes with the OA vs. normal response as reviewed in [26] is also shown for different classes of overlapping proteins. Table S1: List of proteins identified by high-resolution MS/MS of BC-/CS-treated and pCTR human OA synoviocytes secretomes. Table S2: Mean and standard deviation of the 26 detectable cytokines and chemokines quantified by the Bio-Plex multiplex array system in pCTR, BC- and CS-treated OA synoviocytes secretomes.

## Abbreviations

BC	Biotechnological Chondroitin
CS	Chondroitin Sulfate
ECM	Extracellular Matrix
FDRs	False Discovery Rates
GlcN	Glucosamine
GO	Gene Ontology
MS	Mass Spectrometry
NSAIDs	Non-Steroidal Anti-Inflammatory Drugs
OA	Osteoarthritis
SYSADOA	Symptomatic Slow-Acting Drugs
