# Supplementary material for: Differential Secretome Profiling of Human Osteoarthritic Synoviocytes Treated with Biotechnological Unsulfated and Marine Sulfated Chondroitins
Source: Int J Mol Sci. 2020 May 26;21(11):3746. doi: 10.3390/ijms21113746 (PMC7312545; doi:10.3390/ijms21113746)
Supplement: Supplementary file 1 [file ijms-21-03746-s001.zip › Supplementary materials/Figure S1_S4.pptx]

## Slide 1
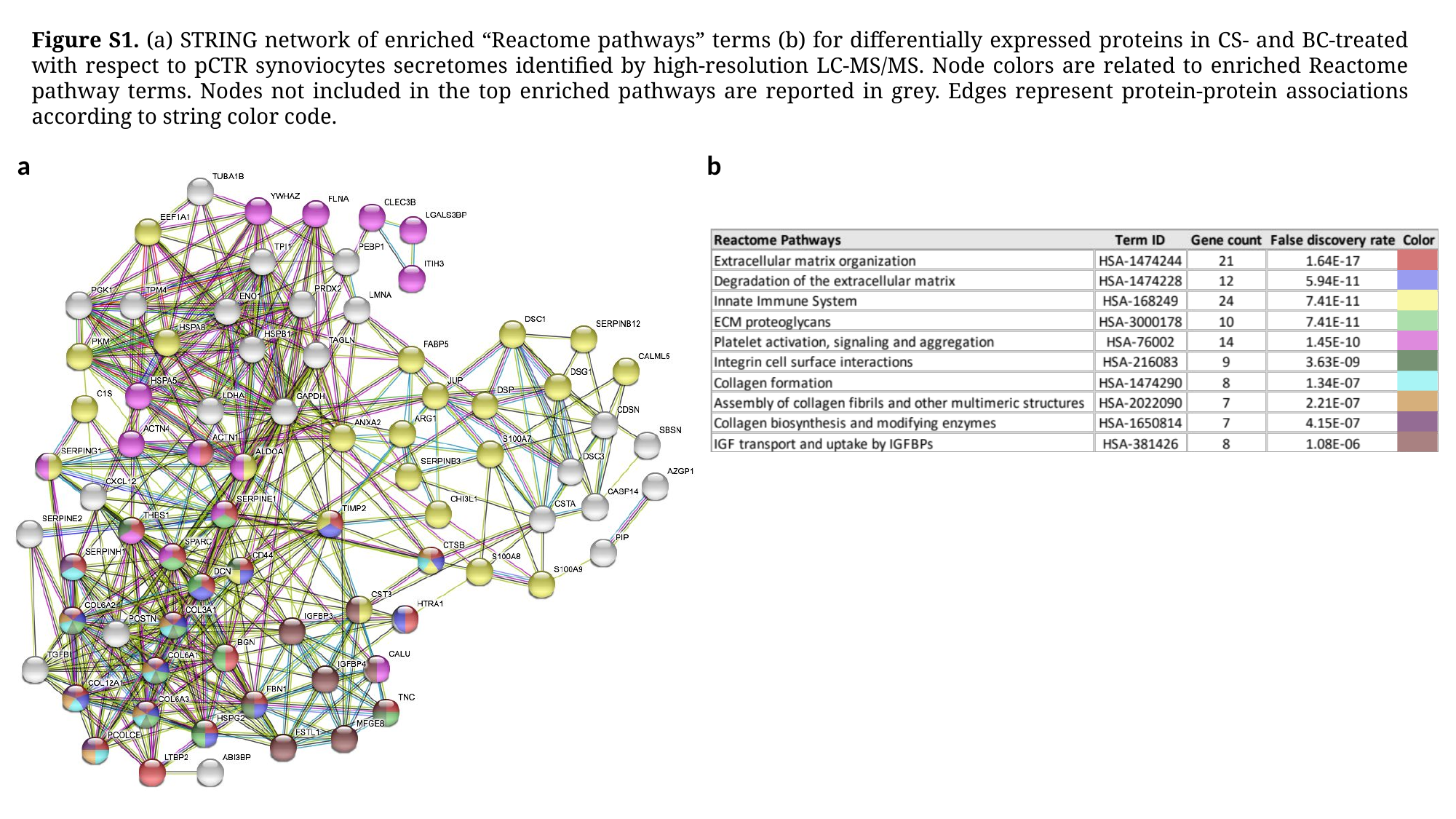

Figure S1. (a) STRING network of enriched “Reactome pathways” terms (b) for differentially expressed proteins in CS- and BC-treated with respect to pCTR synoviocytes secretomes identified by high-resolution LC-MS/MS. Node colors are related to enriched Reactome pathway terms. Nodes not included in the top enriched pathways are reported in grey. Edges represent protein-protein associations according to string color code.
a
b

## Slide 2
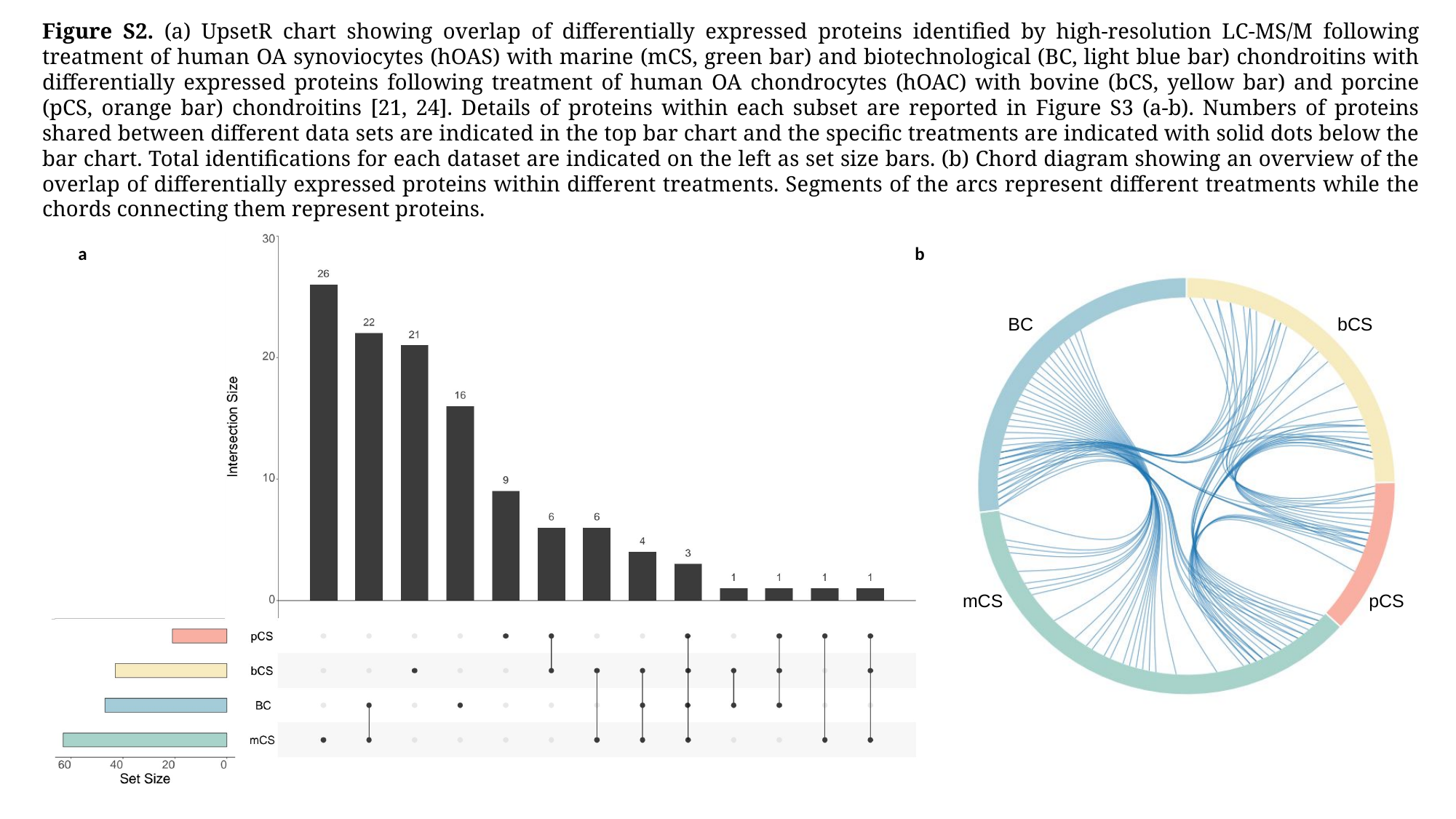

Figure S2. (a) UpsetR chart showing overlap of differentially expressed proteins identified by high-resolution LC-MS/M following treatment of human OA synoviocytes (hOAS) with marine (mCS, green bar) and biotechnological (BC, light blue bar) chondroitins with differentially expressed proteins following treatment of human OA chondrocytes (hOAC) with bovine (bCS, yellow bar) and porcine (pCS, orange bar) chondroitins [21, 24]. Details of proteins within each subset are reported in Figure S3 (a-b). Numbers of proteins shared between different data sets are indicated in the top bar chart and the specific treatments are indicated with solid dots below the bar chart. Total identifications for each dataset are indicated on the left as set size bars. (b) Chord diagram showing an overview of the overlap of differentially expressed proteins within different treatments. Segments of the arcs represent different treatments while the chords connecting them represent proteins.
a
b
BC
bCS
mCS
pCS

## Slide 3
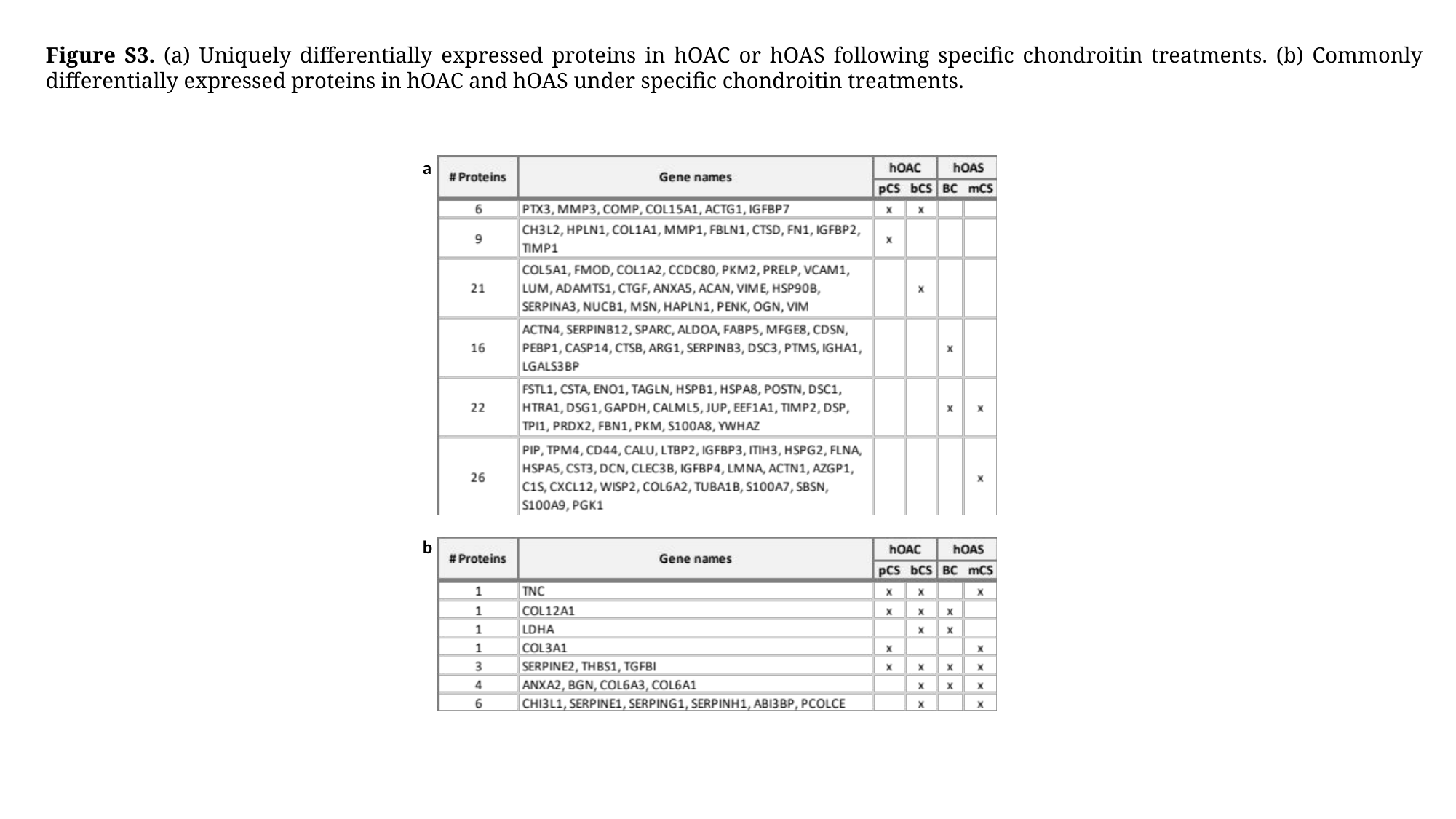

Figure S3. (a) Uniquely differentially expressed proteins in hOAC or hOAS following specific chondroitin treatments. (b) Commonly differentially expressed proteins in hOAC and hOAS under specific chondroitin treatments.
a
b

## Slide 4
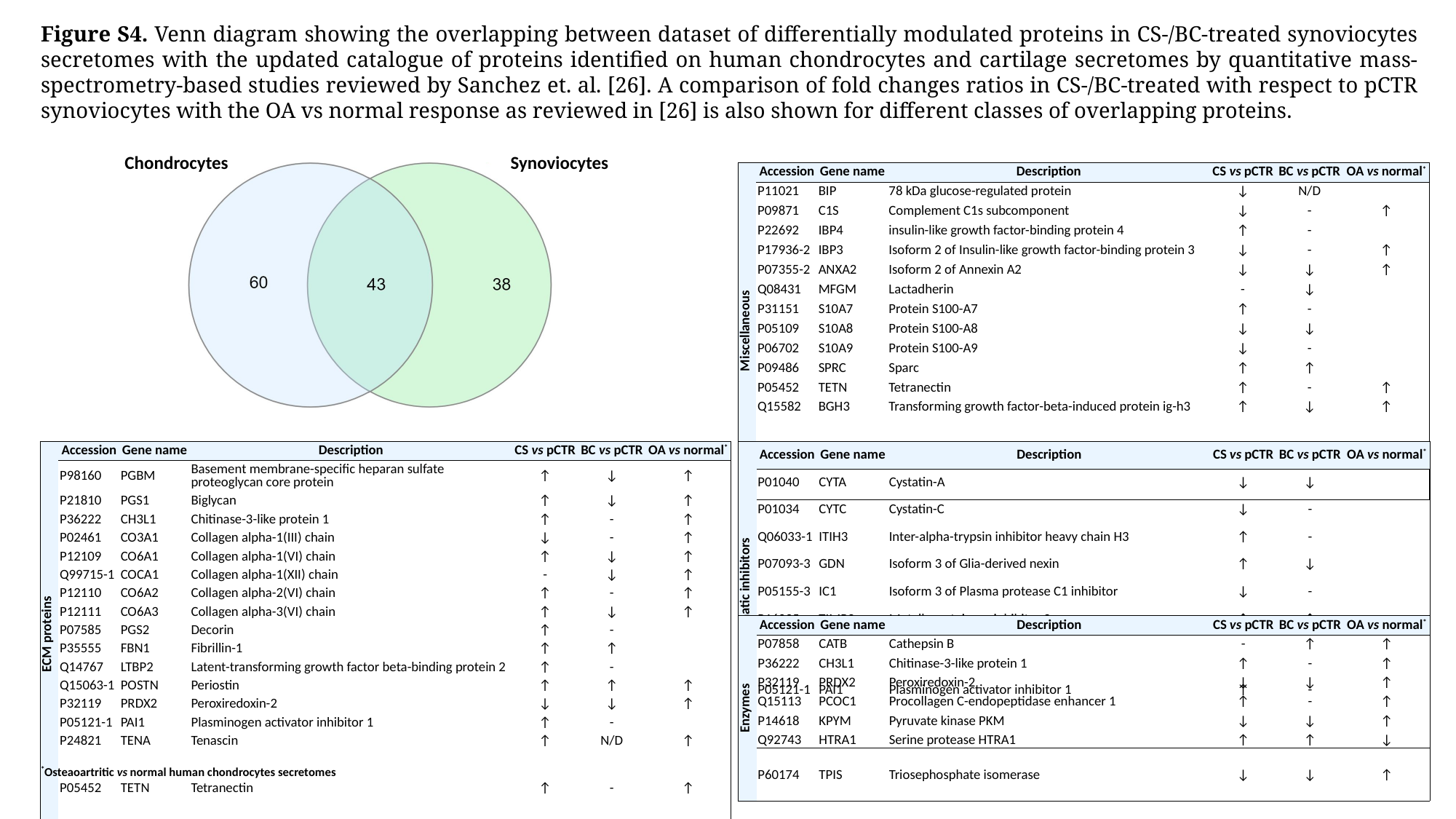

Figure S4. Venn diagram showing the overlapping between dataset of differentially modulated proteins in CS-/BC-treated synoviocytes secretomes with the updated catalogue of proteins identified on human chondrocytes and cartilage secretomes by quantitative mass-spectrometry-based studies reviewed by Sanchez et. al. [26]. A comparison of fold changes ratios in CS-/BC-treated with respect to pCTR synoviocytes with the OA vs normal response as reviewed in [26] is also shown for different classes of overlapping proteins.
B
Chondrocytes
Synoviocytes
| Miscellaneous | Accession | Gene name | Description | CS vs pCTR | BC vs pCTR | OA vs normal\* |
| --- | --- | --- | --- | --- | --- | --- |
| | P11021 | BIP | 78 kDa glucose-regulated protein | ↓ | N/D | |
| | P09871 | C1S | Complement C1s subcomponent | ↓ | - | ↑ |
| | P22692 | IBP4 | insulin-like growth factor-binding protein 4 | ↑ | - | |
| | P17936-2 | IBP3 | Isoform 2 of Insulin-like growth factor-binding protein 3 | ↓ | - | ↑ |
| | P07355-2 | ANXA2 | Isoform 2 of Annexin A2 | ↓ | ↓ | ↑ |
| | Q08431 | MFGM | Lactadherin | - | ↓ | |
| | P31151 | S10A7 | Protein S100-A7 | ↑ | - | |
| | P05109 | S10A8 | Protein S100-A8 | ↓ | ↓ | |
| | P06702 | S10A9 | Protein S100-A9 | ↓ | - | |
| | P09486 | SPRC | Sparc | ↑ | ↑ | |
| | P05452 | TETN | Tetranectin | ↑ | - | ↑ |
| | Q15582 | BGH3 | Transforming growth factor-beta-induced protein ig-h3 | ↑ | ↓ | ↑ |
| | Q01995 | TAGL | Transgelin | ↑ | ↑ | |
| ECM proteins | Accession | Gene name | Description | CS vs pCTR | BC vs pCTR | OA vs normal\* |
| --- | --- | --- | --- | --- | --- | --- |
| | P98160 | PGBM | Basement membrane-specific heparan sulfate proteoglycan core protein | ↑ | ↓ | ↑ |
| | P21810 | PGS1 | Biglycan | ↑ | ↓ | ↑ |
| | P36222 | CH3L1 | Chitinase-3-like protein 1 | ↑ | - | ↑ |
| | P02461 | CO3A1 | Collagen alpha-1(III) chain | ↓ | - | ↑ |
| | P12109 | CO6A1 | Collagen alpha-1(VI) chain | ↑ | ↓ | ↑ |
| | Q99715-1 | COCA1 | Collagen alpha-1(XII) chain | - | ↓ | ↑ |
| | P12110 | CO6A2 | Collagen alpha-2(VI) chain | ↑ | - | ↑ |
| | P12111 | CO6A3 | Collagen alpha-3(VI) chain | ↑ | ↓ | ↑ |
| | P07585 | PGS2 | Decorin | ↑ | - | |
| | P35555 | FBN1 | Fibrillin-1 | ↑ | ↑ | |
| | Q14767 | LTBP2 | Latent-transforming growth factor beta-binding protein 2 | ↑ | - | |
| | Q15063-1 | POSTN | Periostin | ↑ | ↑ | ↑ |
| | P32119 | PRDX2 | Peroxiredoxin-2 | ↓ | ↓ | ↑ |
| | P05121-1 | PAI1 | Plasminogen activator inhibitor 1 | ↑ | - | |
| | P24821 | TENA | Tenascin | ↑ | N/D | ↑ |
| | P05452 | TETN | Tetranectin | ↑ | - | ↑ |
| Enzymatic inhibitors | Accession | Gene name | Description | CS vs pCTR | BC vs pCTR | OA vs normal\* |
| --- | --- | --- | --- | --- | --- | --- |
| | P01040 | CYTA | Cystatin-A | ↓ | ↓ | |
| | P01034 | CYTC | Cystatin-C | ↓ | - | |
| | Q06033-1 | ITIH3 | Inter-alpha-trypsin inhibitor heavy chain H3 | ↑ | - | |
| | P07093-3 | GDN | Isoform 3 of Glia-derived nexin | ↑ | ↓ | |
| | P05155-3 | IC1 | Isoform 3 of Plasma protease C1 inhibitor | ↓ | - | |
| | P16035 | TIMP2 | Metalloproteinase inhibitor 2 | ↑ | ↑ | |
| | P05121-1 | PAI1 | Plasminogen activator inhibitor 1 | ↑ | - | |
| Enzymes | Accession | Gene name | Description | CS vs pCTR | BC vs pCTR | OA vs normal\* |
| --- | --- | --- | --- | --- | --- | --- |
| | P07858 | CATB | Cathepsin B | - | ↑ | ↑ |
| | P36222 | CH3L1 | Chitinase-3-like protein 1 | ↑ | - | ↑ |
| | P32119 | PRDX2 | Peroxiredoxin-2 | ↓ | ↓ | ↑ |
| | Q15113 | PCOC1 | Procollagen C-endopeptidase enhancer 1 | ↑ | - | ↑ |
| | P14618 | KPYM | Pyruvate kinase PKM | ↓ | ↓ | ↑ |
| | Q92743 | HTRA1 | Serine protease HTRA1 | ↑ | ↑ | ↓ |
| | P60174 | TPIS | Triosephosphate isomerase | ↓ | ↓ | ↑ |
*Osteaoartritic vs normal human chondrocytes secretomes
